# The gut microbiome in graft-versus-host disease: mechanisms of immune modulation and therapeutic approaches

**DOI:** 10.1080/19490976.2026.2631224

**Published:** 2026-02-18

**Authors:** Adonai Blessington Moses, Albert C. Yeh

**Affiliations:** a Department of Microbiology, University of Washington, Seattle, WA, USA; b Translational Science and Therapeutics Division, Fred Hutchinson Cancer Center, Seattle, WA, USA; c Division of Hematology and Oncology, University of Washington, Seattle, WA, USA

**Keywords:** Graft-versus-host disease, GVHD, stem cell transplantation, bone marrow transplantation, gut microbiota, microbiome

## Abstract

Graft-versus-host disease (GvHD) remains a major complication of allogeneic hematopoietic stem cell transplantation and occurs when T cells from the donor graft target recipient-derived antigen on host tissue. The involvement of the gastrointestinal (GI) tract drives morbidity and mortality—not coincidentally, the GI tract also harbors the most complex and abundant human microbial reservoir. In this review, we first revisit how the microbiota initiates, propagates, and protects against GvHD in the context of both innate and adaptive immunity. Historically, the impact of the microbiota on GvHD has been ascribed primarily to the activation of innate immunity, setting the stage for donor alloreactivity. Although established models of GvHD focus on donor–host genetic disparity as the principal driver of donor T-cell activation, commensal microbes in the GI tract, whose collective gene content exceeds that of the human genome by more than two orders of magnitude, constitutes an immense and poorly understood source of potential T-cell antigens. We next discuss the evolution of therapeutic approaches aimed at modifying the microbiota to improve GvHD outcomes, incorporating over 40 clinical studies spanning the last 40 years, from broad decontamination strategies to pre/probiotic approaches and targeted ecosystem replacement, including fecal microbiota transplantation.

## Introduction

Allogeneic hematopoietic stem cell transplantation (HSCT) represents a curative approach for a range of hematologic malignancies. However, its clinical use is often limited by graft-versus-host disease (GvHD), a potentially life-threatening complication that results from major and minor histocompatibility mismatches between the donor and host,^1^ culminating in the attack of host tissue by the adaptive immune response of the donor. Involvement of the gastrointestinal (GI) tract represents the most morbid form of acute GvHD,^2^ and initial observations over half a century ago demonstrated that depletion of the GI flora in gnotobiotic mice significantly decreased GI GvHD.^3^^,^^4^ Early enthusiasm for GI decontamination as a preventative approach led to several single-center clinical studies in the 1980s and 1990s that demonstrated the potential impact of environmental decontamination including the use of oral antibiotics to reduce GvHD risk.^5-8^ However, with the adopotion of calcineurin inhibitiors to prevent GvHD, the practical difficulty of achieving strict decontamination in the clinical setting, lack of nuanced mechanistic insight and limited ability to examine individual microbial taxa, and push for antibiotic stewardship hindered sustained adoption of using broad spectrum antibiotics as a preventative measure.

With the increasing availability of bacterial genomic sequencing in the mid-2000s, the initial brute force approach of depleting the microbiota as a single entity would yield to the concept of preventing dysbiosis, characterized by the loss of apparently beneficial, often anaerobic commensal bacteria associated with favorable GvHD outcomes^9-12^ and expansion of pathobionts associated with increased GvHD.^13-15^ Broad GI decontamination that impact anaerobic bacteria was thought to reduce beneficial commensal organisms,^16^ and the emphasis shifted away from antibiotic utilization toward selective microbial reconstitution. A 2020 landmark study with the largest patient cohort to date—examining 8,767 fecal samples from 1,362 patients undergoing allogeneic transplant—highlighted this concept and demonstrated that a reduction of alpha diversity in the GI microbiota and domination of specific taxa at the time of neutrophil engraftment were linked to GvHD mortality. The challenge now became clarifying cause–effect relationships of individual bacterial taxa's effects on outcomes. Additional studies examining specific enzymatic^11^^,^^13^^,^^15^ and metabolic pathways^9^^,^^17^^,^^18^ have identified novel mechanisms by which individual microbes mediate or protect from GvHD. Modern clinical approaches emphasize targeted microbial interventions aimed at preserving or expanding beneficial commensal anaerobes and include the utilization of narrow-spectrum antibiotics,^19^ prebiotic support,^20^^,^^21^ and post-transplant microbiota manipulation, including fecal-microbiome transplantation,^22-25^ to mitigate GvHD risk.

Despite extensive preclinical insight and efforts at clinical translation over the past several decades, however, standard practice guidelines for GvHD prophylaxis in the United States and Europe do not currently recommend routine modulation of the GI microbiota,^26^^,^^27^ reflecting the complexity in deconvoluting the multiple mechanisms that co-exist in promoting or reducing GvHD risk as well as therapeutic challenges in implementing and sustaining targeted microbiota changes. In this review, we summarize recent mechanistic insights into how the microbiota shapes GvHD pathogenesis as well as current therapeutic strategies, highlighting the need to understand the microbiota composition in the context of donor and recipient immunity.

## Microbial mechanisms of immune modulation

**Figure 1.**
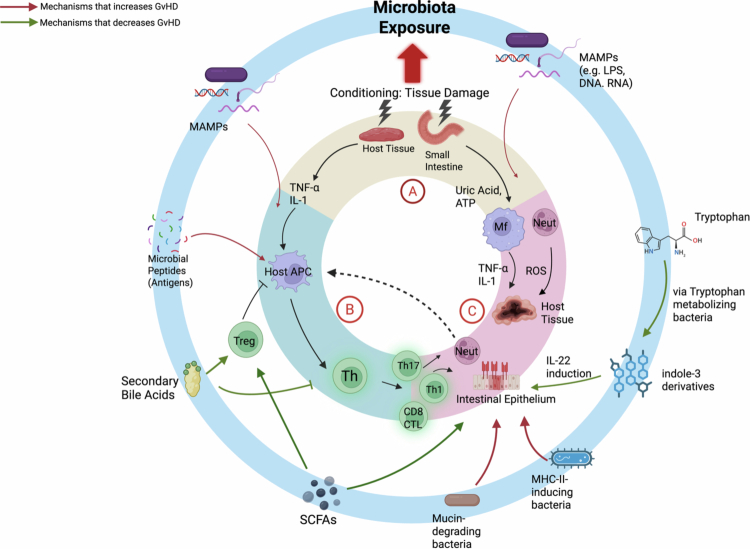
GvHD pathogenesis emphasizes the microbiota‒immune axis. This diagram augments the classic three-step model of GvHD pathophysiology proposed by Ferrara et al.: conditioning-induced epithelial injury, antigen presentation, and the donor T-cell response, within the context of microbiota exposure. (A) Phase I: tissue damage from conditioning releases uric acid, ATP, and other danger signals that, together with microbial-associated molecular patterns (MAMPs), activate host antigen-presenting cells (APCs), and inflammatory myeloid cells through TLR and NLRP3 signaling. (B) Phase II: activated APCs present both host alloantigen and microbial antigens, while microbial metabolites—including short-chain fatty acids (SCFAs), secondary bile acids, indole derivatives, and tryptophan catabolites—modulate the differentiation of Th1, Th17, and Treg subsets. (C) Phase III: tissue injury is amplified by ongoing microbial translocation and loss of epithelial barrier integrity, with mucous- and junction-degrading taxa further promoting inflammation. The outer ring (light blue) highlights representative microbial functions that modulate each phase, including SCFA producers that support epithelial integrity and promote Treg expansion along with secondary bile acids, tryptophan-metabolizing bacteria that induce IL-22 via AhR, mucin-degrading organisms that weaken the epithelial barrier, and MHC class II–inducing taxa that enhance antigen presentation. Green arrows denote pathways associated with protection from GvHD, while red arrows indicate mechanisms that exacerbate disease. Adapted from Ferrara et al. (28).

The broad impact that microbial organisms have on acute GvHD is illustrated by augmenting the widely cited three-step model of acute GvHD pathophysiology proposed by Ferrara et al.^28^ While this decades-old paradigm has primarily attributed the role of the microbiota in activating the innate immune response (phase 1), we now understand that the microbiota affects all three phases in the GvHD pathway, including stimulating antigen presentation, shaping the T-cell response, and exacerbating host tissue injury (Figure 1).

### Phase 1: microbial exposure and the innate immune response

Cytotoxic conditioning prior to transplantation remains critical for successful donor cell engraftment and prevention of graft rejection but also results in tissue damage. The subsequent release of damage-associated molecular patterns (DAMPs) and exposure to microbial-associated molecular patterns (MAMPs) is central to the pathogenesis of GvHD and the initiation of the innate immune response^28^ (Figure 1A). Evidence that the gastrointestinal microbiota can impact tissue integrity following total body irradiation (TBI) was reported as early as the 1960s, when work in murine models demonstrated that the lifespan of the intestinal epithelium is reduced in conventional vs. germ-free mice.^29^ Mechanistic insight gleaned from murine experiments in the 1990s demonstrated that tissue injury following TBI enabled the translocation of lipopolysaccharide (LPS), a prototypical MAMP, from damaged intestinal mucosa, which directly amplified GvHD. Conditioning regimen intensity augments the host innate immune response by increasing macrophage production of tumor necrosis factor alpha (TNF),^5^ which is further amplified by LPS exposure^30^^,^^31^ via toll-like receptor (TLR) 4 signaling.^32^ Exposure to LPS also drives donor cell production of TNF, as the utilization of LPS-resistant donor grafts results in diminished GvHD.^33^

Concurrently, other MAMPs were identified that activate the innate immune response through pattern recognition receptors^34^ including the cell wall components lipoteichoic acid and peptidoglycan, which activate TLR2^35^^,^^36^ and intracellular stimulation of NOD1/2,^37^^,^^38^ flagellin/TLR5,^39^ and CpG-containing DNA/TLR9.^40^ Commensal bacteria subsequently drive the expression of IL-1β, a potent immune-stimulatory cytokine, which lies downstream of the TLR/NF-kB axis.^41^ IL-1β is expressed predominantly by host dendritic cells (DCs) early post-transplant followed by donor DCs, and its production was abrogated following gut decontamination prior to transplant. DNA-mimicking CpG substrates also accelerated GvHD via TLR9 ligation of host antigen-presenting cells^42^ and were not shown to have an effect on TLR9- or MyD88-knockout mice. Neutrophil infiltration in the ileum following transplantation was dependent on local microbial flora and was absent in germ-free mice.^43^ In contrast to the introduction of wild-type neutrophils, TLR-deficient neutrophils mitigated GvHD severity. Similarly, gut decontamination prevented the ability for neutrophils to migrate to mesenteric lymph nodes (mLNs) and activate CD4 T cells in a MHCII-peptide dependent manner.^44^

Exposure to MAMPs during the conditioning phase of HSCT provided the initial biological insight for how microbial decontamination resulted in decreased GvHD risk. While there is strong evidence that this pathway plays a critical role in GvHD initiation, total sterilization, while compelling in theory, remains impractical. A modern understanding of the GI microbiota also suggests that protective factors attributed to specific bacterial taxa would be lost to broad decontamination. Nonetheless, in the absence of mitigating factors, MAMP exposure offers a clear pathway to explain why domination of certain pathobionts, such as *Escherichia coli* may aggravate GvHD.

### Phase 2: adaptive immunity—how microbes shape the donor T-cell response

**Figure 2.**
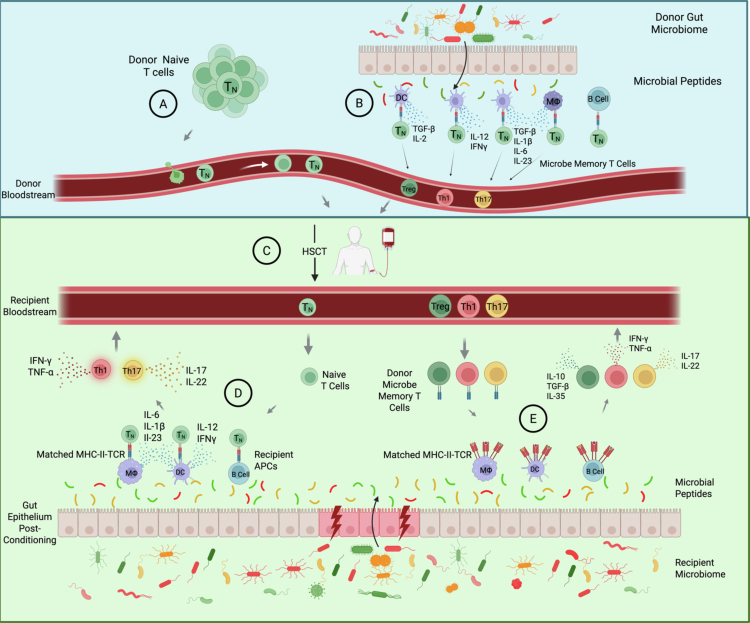
Donor and recipient microbiota modulate the adaptive T-cell response. Naïve T cells that potentially recognize cognate microbial antigens in the recipient as well as microbe-specific memory T cells are found in the donor graft. The resulting impact in the recipient is illustrated here, assuming an MCH-matched setting. In the MHC-mismatched setting, the naïve T-cell compartment plays a more significant role. (A) Naïve T cells shown here have the potential ability to recognize microbial peptides in transplant recipients. (B) Donor microbiota–derived peptides promote Th1 (red), Th17 (yellow), or Treg (green) polarization of donor memory T cells prior to transplantation. While initially primed in the gastrointestinal tract, these microbe-specific memory T cells can also be found in the peripheral blood and thus constitute part of the donor graft. (C) Transfer of donor naïve and microbe-specific memory T cells to the recipient. (D) Conditioning-induced mucosal injury enables access to recipient microbiota peptides presented by host antigen-presenting cells (APCs), driving the de novo differentiation of naïve T cells into pathogenic Th1 and Th17 cells due to the proinflammatory cytokine environment characterized by elevated levels of IL-12, IL-1beta, IL-6, and IFNγ. (E) Microbe-specific memory T cells rapidly exert effector functions, including pro- and anti-inflammatory responses, upon recognition of cognate antigens in the proper MHC II context, with the resulting cytokine milieu depending on initial priming in the donor.

While the classic GvHD paradigm emphasizes genetic mismatch (either major or minor antigen differences between donor and host) as the source of donor T-cell reactivity, this underestimates the potential pool of antigens presented to donor T cells (Figure 1B). An estimated 10^12^ commensal microbes reside in the human gastrointestinal tract,^45^ and insights over the past two decades have proven that microbes not only shape how T cells are polarized but also dictate the clonotypic response in the gastrointestinal tract during homeostasis, infection, and inflammation.^46-54^ However, much of the understanding of T-cell responses to microbes has been derived outside of the HSCT context. Allogeneic transplantation is unique due to the introduction of donor T cells, which are primed by the donor but not the recipient microbiota. Recent evidence has demonstrated that the recognition of microbial peptides can directly impact GvHD, as the expansion of microbiota-specific T cells from the donor graft, which target MHC II peptides derived from bacterial flagellin or *Clostridium savagella* in the recipient GI tract, exacerbated GvHD in murine models harboring respective commensal organisms.^46^ How donor T cells respond to recipient microbial antigens in the clinical setting and the subsequent impact on clinical outcomes remain poorly understood. Here,we propose a framework in thinking about how microbes shape the donor T-cell response by first examining microbiota-reactive T cells in the donor graft (Figure 2).

#### Donor memory T cells are shaped by the donor microbiota

Peripheral blood CD4 T-cell reactivity to bacterial species is normal and has been demonstrated in both murine and human context covering multiple microbial taxa.^53^^,^^55^^,^^56^ Microbiota-specific memory T cells in circulation that react to heat-inactivated *Escherichia, Lactobacillus, Bifidobaterium, Faecalibacterium, Bacteroides, Roseburia, Ruminococcus, Salmonela,* and *Clostridium* comprising up to 0.4% of CD4^+^ T cells per taxa have been reported in healthy inividuals.^55^ Phenotypic diversity in this setting can also be seen by a broad range of T-cell responses to microbial peptides, in which numerous predicted bacterial epitopes were identified to elicit a combination of IFNγ, IL-10, or IL-17 responses from 40 patient peripheral blood samples.^56^

Following introduction of the donor graft in HSCT, we posit that microbiota-specific memory T cells may thus play a protective or pathogenic role depending on whether they encounter cognate peptide presented on the proper MHC II allele and their polarization state (Figure 2). For the first to occur, the cognate donor MHC II allele must also be present in the recipient. Second, T-cell polarization depends on the donor environment and exposure to specific microbes. For example, anaerobic bacteria such as *Faecalibacterium prausnitzii* that produce short-chain fatty acids (SCFA) experimentally induce Treg differentiation,^57^ and IL-10 producing CD4^+^ T cells have been identified reactive to *F. prausnitzii* in human peripheral blood.^55^ Thus, a donor graft containing *F. Prausnitzii*-specific T cells could play an immunosuppressive/protective role in GvHD biology if they encounter cognate antigens in the recipient microbiota. Clinically, this hypothesis is consistent with the observation that reconstitution of commensal anaerobes known to produce SCFA appears to reduce GvHD risk. Additionally, the optimal donor with regards to harboring regulatory microbiota-specific T cells may be the one with the most similar commensal microbiota composition in the proper HLA context (e.g. matched transplant), as this would maximize the chance of cognate recognition.

#### Naïve donor T-cell recognition of microbial peptides is likely to promote GVHD during HSCT

The majority of donor T cells are naïve, and a subset of these could be activated by commensal microbial peptides in the recipient in an MHC-matched or mismatched context. As the peri-transplant phase is characterized by excess inflammatory cytokine production, including IL-12, IFNγ, and IL-6 from macrophages, DCs, and other alloreactive T cells, in response to tissue injury and DAMP/MAMP exposure, naïve T cells that recognize cognate microbial antigens are most likely to be skewed toward Th1 or Th17 phenotypes that contribute to GvHD pathology (Figure 2).

This hypothesis is well-supported by evidence outside of the HSCT literature. T-cell mediated tolerance to commensal organisms is contextual, as proinflammatory cues shift the balance towards a pathogenic response. Acute gastrointestinal infection with orally administered *Toxoplasma gondii* elicited the activation and Th1 differentiation of flagellin-targeting intestinal T cells directed at commensal *Clostridium XIVa* bacteria that persist as long-term memory T cells.^47^ Peripheral induction of colonic Tregs with TCR repertoires distinct from those of Tregs of other tissue types was demonstrated for *Clostridiales* and *Paraacteroides*, for example, although the same Treg clonotypes, when adoptively transferred predominantly as Foxp3^−^ T cells, induced experimental colitis in Rag1^−/−^ hosts.^58^ Similarly, the transfer of *Helicobacter hepaticus*-specific T cells induced RORγT^+^ Foxp3^+^ Tregs in the colon of *H. hepaticus* colonized wild-type mice but transfer of the same cells into IL-10^−/−^ mice or mice exposed to an IL-10 blockade antibody resulted in the generation of microbiota-specific Th17 cells that drive severe colitis.^50^ In a CD4^+^ T-cell driven model of allogeneic transplantation, the cotransfer of H-Y-targeting (alloreactive) donor T cells into male hosts augmented IFNγ expression of transferred flagellin-targeting CD4^+^ T cells.^46^ In the clinical setting, SusC, a membrane protein epitope derived from the *Bacteroides* genus, elicits an IL-10 tolerant response from CD4^+^ T cells in healthy individuals. However, during Crohn's disease, T-cell responses toward SusC shift to a proinflammatory Th17 phenotype.^59^

From a clinical perspective, it would be logical to therefore minimize naïve T-cell recognition of cognate microbial antigens, a hypothesis consistent with the observation that MHC-mismatched transplants carry a higher risk of GvHD. For MHC-matched transplants, exposure of naïve donor T cells to novel microbial antigens in the recipient risk pathologic microbial T-cell expansion. With improvements in GvHD outcomes and prophylaxis,^60^ particularly with regimens that blunt the initial expansion and activation of alloreactive donor T cells across both donor-recipient matched and mismatched settings,^61-64^ the microbial-driven donor T-cell response may play an increasingly significant role in restoring immune homeostasis in the intestinal tract.

### Phase 3: impact of the microbiota on host tissue injury and susceptibility

The final effector phase of GvHD pathogenesis amplifies local tissue injury, further propagating the underlying inflammatory cycle.^28^ Several microbial-related mechanisms have been shown to contribute to host tissue injury or protection (Figure 1C). Microbial byproducts such as LPS that stimulate macrophage-dependent TNF release can directly result in tissue injury.^65^ Metalloprotease secretion found in taxa such as *Enterococcus*, which can degrade tight junction proteins and increase intestinal permeability,^13^^,^^66^ can also increase susceptibility to tissue injury. Microbe-derived bile acids that mitigate farnesoid X receptor activation was shown to inhibit proliferation and cytokine production of donor T cells in pre-clinical transplant models, and GVHD patients harbored a reduction in microbe-derived bile acids upon disease onset.^67^

Understanding the spatialtemporal axis of exposure to microbial byproducts along the cycle of GvHD is needed for effective clinical translation. Production of butyrate, a microbial SCFA metabolite that is particularly abundant in *Clostridia* has perhaps received the most attention for promising its ability to preserve epithelial cell integrity as an energy source for enterocytes^12^^,^^68^ and induce intestinal Tregs,^69^ which have been recently reviewed.^70^^,^^71^ However, butyrate can also suppress intestinal stem cells proliferation and its potentially beneficial effect blunted if exposed to the intestinal crypts.^72^ Microbial catabolism of dietary tryptophan generates indole derivatives that activate the aryl hydrocarbon receptor (AhR), a transcription factor that promotes IL-22 production by innate lymphoid and T cells to enhances epithelial regeneration and barrier repair.^73^ Preclinical models suggested that absence of the enzyme indoleamine 2, 3-dioxygenase worsened GvHD and augmented the donor T-cell response, but production of urinary indole metabolites have also been associated with worse GvHD outcomes in a study of 54 patients^74^ and was correlated with steroid-refractory disease.

Another major challenge that confronts studying complex microbial ecosystems in GvHD is understanding how individual bacterial mechanisms that influence susceptibility to tissue injury interact in concert. Recent studies have demonstrated that degradation of the protective mucus layer in the intestinal tract provides another key pathway for aggravating GvHD. *Bacteroides thetaiotaomicron*, which expands in the gastrointestinal tract following treatment with carbapenem antibiotics,^15^ upregulates the ability to degrade mucin glycans and thins the colonic mucosal layer, increasing susceptibility to intestinal injury.^15^ Similarly, *Akkermansia muciniphilia,* another commensal organism that also possesses mucus-degrading capabilities, expanded following antibiotic treatment in murine models and was correlated with clinical GvHD.^75^ Conversely, the beneficial effects of *Bacteroides ovatus* were linked to its ability to metabolize dietary polysaccharides, which suppress the mucus-degrading capabilities of other bacteria, including *B. thetaiotaomicron*and *A. muciniphilia* and reduce GvHD-related mortality.^11^

Compounding the complexity of this process, the tug-of-war between individual microbial species exist can vary across multiple phenotypic axes. For example, bacteria taxa increases sensitivity to GvHD by inducing MHC class II expression in the intestinal epithelium, while other taxa can suppress MHC class II expression.^76^ MHC class II inducers include well-studied species such as *C. savagella* in murine models that would align with previously observed phenotypes. However, *B. thetaiotaomicron* in this context was shown to suppress epithelial MHC class II expression and therefore diminish the risk of tissue injury, contrary to its role as a mucin-degrader.

Clinically, it would be most practical to focus on reconstituting the posttransplant microbiota based on the functional classification of taxa that maximize beneficial effects (e.g. SCFA producers) and minimize known detrimental effects such as the ability to degrade mucin or augment the antigen presentation machinery, as exemplified above. Rather than focusing on reconstituting individual microbial strains where durable engraftment can often pose a challenge, a functional emphasis provides a strong rationale for community-based microbial reconstitution. This approach forms the cornerstone of modern microbiota therapeutics that replace or augment “beneficial” bacterial ecosystems via defined bacterial consortia, third party/healthy donor fecal microbiota transplantation, and pre/postbiotic therapies.

## The evolution of interventional approaches targeting the gut microbiota in GvHD

**Figure 3.**
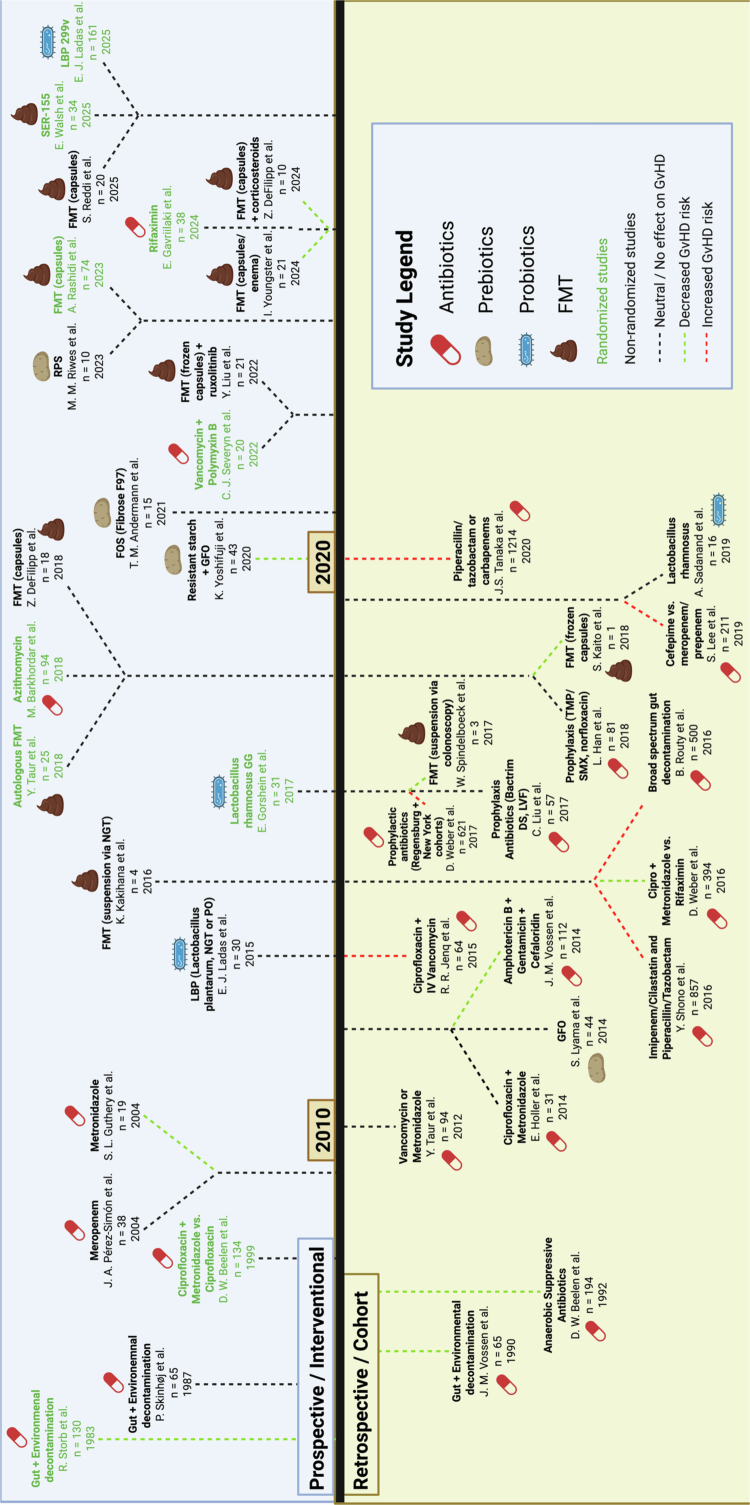
Microbiota-targeted interventions in allogeneic stem cell transplantation. This timeline summarizes the evolving landscape of microbiome-targeted interventions evaluated in allo-HSCT, spanning antibiotics, prebiotics, probiotics, and fecal microbiota transplantation (FMT). The upper half includes prospective, interventional studies while the bottom half comprises retrospective/observational studies. Early approaches focused on broad microbial decontamination, while recent studies emphasized microbiome restoration and precision modulation to mitigate graft-versus-host disease (GvHD). Green text denotes prospective randomized studies. The black dashed lines connecting the study to the timeline indicate no observable impact on GvHD vs. the green dashed line (decrease in GvHD) and the red dashed line (increase in GvHD). Additional details about each study can be found in Supplemental Table 1.

As recent reviews have already discussed many of the key observational and interventional studies in the field,^70^^,^^71^^,^^77-79^ we focus on integrating findings in the broader context and presents three main types of interventions that have evolved over time: 1) decontamination strategies, 2) pre/postbiotic usage, and 3) ecosystem restoration (Figure 3). Some of the key challenges, particularly over the previous decade, include the lack of prospective studies and reliance on retrospective observations, relatively small sample sizes for interventional studies, and heterogeneity in treatment approaches even within the same interventional category. Notably, over the past several decades, there has been a shift from decontamination approaches, including antibiotic regimens, towards augmenting or replacing the commensal ecosystem following transplant (Figure 3, pre/postbiotics and FMT). We discuss the main ideas driving this shift below.

### Part 1: microbial decontamination

Initial studies that demonstrated reduced GvHD risk in germ-free animal models^3^^,^^4^ naturally led to the adoption of decontamination strategies to improve GvHD outcomes. Strict protocols employing techniques such as laminar and reverse flow isolation rooms, sterilized food, broad-spectrum nonabsorbable oral antibiotics, and skin cleansing in the peritransplant window did appear to reduce but did not eliminate acute GvHD and infectious risk.^5-7^^,^^80^^,^^81^ Targeted cultures demonstrated effective, but not always complete microbial suppression.^80^ Decontamination in the clinical setting proved significantly more difficult to achieve compared to the preclinical setting, and antibiotic usage, particularly those with broad anaerobic activity, such as piperacillin/tazobactam and carbapenems, was also observed to paradoxically increase GvHD risk,^16^^,^^82-84^ questioning the wisdom of this approach. Further improvement in bacterial classification via identification with 16S rRNA sequencing led to the realization that commensal anaerobes, which were previously difficult to isolate, were associated with reduced GvHD mortality.^12^ Bacterial diversity, which closely reflects the collective state of commensal anaerobes and is susceptible to broad antibiotic exposure, was often adopted as a surrogate clinical endpoint owing to the correlation between lower diversity, unfavorable GvHD outcomes, and expansion of pathobionts such as *Enterococcus* and *Escherichia.*^14^

The use of selective antibiotics outside of standard gram-negative prophylaxis may be beneficial for GvHD outcomes but has not been conclusively proven. For example, in a retrospective study of 394 consecutively treated patients, rifaximin, when used for gut decontamination starting 8 days before transplant and continuing until 14 days after engraftment, correlated with lower enterococcal positivity, higher urinary 3-indoxyl sulfate associated with commensal bacteria, and lower 1-year transplant related mortality compared to ciprofloxacin/metronidazole.^19^ However, rifaximin has also been shown to breed resistant enterococcal species,^85^ and a recent prospective study enrolling 38 patients comparing rifaximin vs. ciprofloxacin for neutropenic prophylaxis did not demonstrate statistical difference in the incidence of grade 2+ aGvHD (rifaximin 44.4% vs. 60%).^86^ Overall, antibiotic usage to improve GvHD outcomes has been controversial, and current practices have shifted toward selectively using broad-spectrum antibiotics only when necessary for infectious risk, with preservation of microbial diversity and beneficial commensal anaerobes being key arguments for antibiotic stewardship. Approaches to reduce GvHD risk have subsequently focused onselective restoration of a beneficial microbial ecosystem (Figure 3).

### Part 2: pre/probiotics

The fermentation of commensal anaerobes with prebiotics (oral substrates that selectively promote beneficial commensal organisms) or direct delivery of favorable bacterial strains (probiotics) has been explored over the past decade as an alternative approach to antibiotics (Figure 3). Small studies have shown that preservation of mucosal integrity, microbial diversity, and luminal SCFA content may be achieved through enteral supplementation during the peritransplant period, although the definitive improvement in intestinal GvHD outcomes remains unclear. An early retrospective study of 44 patients comparing the impact of ingesting the commercial enteral supplementation product glutamine, fiber, and oligosaccharides (GFO) demonstrated a decreased duration of oral mucositis and a trend towards reduced *Enterococcus* translocation.^87^ Ingestion of resistant starch improved mean fecal butyrate levels in 20 healthy patients, although individual responses varied widely.^88^ A follow-up prospective study including 49 patients demonstrated that oral intake of resistant starch (Amylofiber SH) and a prebiotic mixture containing glutamine, polydextrose and lactosucrose from conditioning until 28 days posttransplant decreased the duration of oral mucositis and reduced grade II-IV GvHD incidence,^89^ although improvement in GvHD outcomes was significant for skin GvHD but not gut and liver GvHD. Secondary analyses suggested that patients taking prebiotics were more likely to have maintained or increased microbial diversity and luminal butyrate concentrations by day 28. In a recent feasibility study, 10 patients undergoing allogeneic transplant received resistant potato starch (RPS) from day −7 to day 100, with significantly higher fecal butyrate levels in participants on RPS.^21^ The corresponding phase II study with a target enrollment of 70 patients and primary outcome of incidence of aGVHD is currently pending (NCT 06784336).

The use of probiotics has yielded mixed results. While murine studies have suggested that the introduction of *Lactobacillus rhamnosus* reduced bacterial translocation and improved GvHD outcomes,^90^ a randomized study of 31 adults comparing *L. rhamnosus* supplementation starting at the time of engraftment to no probiotic exposure did not show a difference in aGvHD incidence nor in *Lactobacillus* colonization, suggesting a lack of effective microbial engraftment.^91^ Another recent large clinical trial (ACCL1633) including 161 patients found that the administration of *Lactiplantibacillus plantarum* from the start of conditioning to 56 days post-transplant period to be safe but ineffective at preventing GI GvHD.^92^ However, there was insufficient data to assess the efficacy of microbial engraftment. If engraftment can be more convincingly achieved, it is possible that probiotics could represent a viable approach with proper bacterial strain selection. A pilot study utilizing stool from patients without GvHD demonstrated strain-to-strain variability in the capacity of *Clostridium bolteae*, *Blautia* spp., and *Bifidobacterium longum* to produce SCFAs,^93^ and that optimization of SCFA production through strain selection could mitigate GvHD more effectively than a standard consortium of the same species in a mismatched murine transplant model.

### Part 3: ecosystem restoration

Fecal microbiota transplantation (FMT) has emerged as a promising therapeutic strategy to improve transplant outcomes and is already well-established as a safe therapeutic option for refractory *Clostridium difficile* infection.^94^ The potential advantage of FMT over pre- and probiotics is the ability to directly deliver desired bacterial species of interest and potentially shorter treatment interval compared to the former, as well as overall superior engraftment rates compared to the latter. Initial case series in patients with steroid-refractory (SR) GI GvHD demonstrated that FMT from healthy adult donors transferred as homogenized stool solution through a colonoscope could lead to a reduction of GI symptoms and ultimately resolution of GvHD but required administration at repeat intervals (up to 6 times reported at interval of 2–3 weeks).^95^ Soon after, it was demonstrated that FMT administered as a frozen capsule could also be effective at restoring bacterial diversity and treating SR aGvHD,^25^ and a larger prospective study treating 21 patients at the time of SR aGvHD diagnosis demonstrated that the restoration of taxa associated with favorable GvHD outcomes (*Clostridiales,* SCFA-producers) alongside a decrease in taxa associated with poor outcomes (*Enterobacteriaceae).*^24^ The trial demonstrated an overall response rate of 52.4%, though sustained responses were rare^24^ and likely could have improved with repeated treatment administration. The combined use of FMT with ruxolitinib to treat patients with steroid-refractory GI GvHD in a small cohort of 21 patients led to a high overall response rate of 71.4% by day 28, with an associated decline in the serum IL-2, IL-17A and fraction of CD69^+^ T cells.^96^ Another study including 10 patients with high-risk, treatment-naive acute lower GI GvHD demonstrated the feasibility and efficacy of using FMT in combination with corticosteroids as initial treatment,^23^ with an organ-specific complete response rate of 70% at day 28 that correlated with the production of metabolites, including tryptophan and SCFAs.

The success of FMT in treating GvHD has led to efforts at employing FMT as a prophylactic measure to improve transplant outcomes, including both infection prevention and reduction in GvHD risk. In a recent prospective randomized trial enrolling 74 patients comparing healthy donor FMT to placebo control FMT delivered as a capsule for 2 days following neutrophil recovery posttransplant, exposure to FMT resulted in a lower 4-month infection density (0.74 vs. 0.91 events per 100 patient days) but numerically increased GvHD risk.^97^ Optimization of FMT also involves judicious donor selection. A preplanned interim analysis revealed a donor-specific effect with respect to microbiota engraftment from three different donors for 20 recipients, as all cases of severe aGvHD were attributed to a single donor.^22^ While a reduction in GvHD risk has not yet been demonstrated for FMT prophylaxis, a reduction in infectious risk highlights several potential positive attributes of FMT: (1) the restoration of colonization resistance by diversifying the anaerobic community and preventing domination by pathogenic taxa, which is a major predictor of bloodstream infection, and (2) the improvement of mucosal immunity through the augmentation of luminal SCFA production and the restoration of the epithelial barrier.

Investigational studies with microbiome therapeutics SER-155 (comprising 16 bacterial *Firmicutes* strains) extend these concepts beyond conventional FMT by delivering a defined consortium of cultivated bacterial strains designed to restore gut microbiota diversity, strengthen epithelial barrier integrity, and reduce bacterial bloodstream infections and GvHD in allo-HSCT patients.^98-100^ Similar to FMT, this type of approach has been shown to be effective at reducing recurrent *C. difficile* infection (SER-109, *Firmicutes* spores comprising 77 bacterial genera).^101^ A phase 1b study of SER-155 demonstrated its safety, durable engraftment of bacterial strains, significant reduction in bloodstream infections by 77%, decreased antibiotic exposure, and lower severity of febrile neutropenia,^98^^,^^99^ with final analysis pending (NCT04995653). Successful engineering of defined consortia to achieve clinical end points has the added benefit of bypassing donor selection variability that poses a significant challenge to FMT delivery.

## Future directions

The collective evidence reviewed here establishes a central principle: the microbiota is not an accessory variable in GvHD, but a core determinant of how epithelial injury, antigen presentation, and donor T-cell programming unfold after transplantation. The convergence of data across epithelial biology, innate sensing, microbial metabolite signaling, and adaptive T-cell response highlights microbial cues that shape both the magnitude and character of the alloreactive response and thus directly influence GvHD severity and clinical outcomes. Microbiome-directed interventions ranging from antibiotic stewardship to FMT and defined bacterial consortia are most effective when they preserve or restore microbial functions rather than target individual taxa, emphasizing the need to transition from the polarized classification of “good” vs. “bad” microbes towards an understanding of protective ecological and metabolic activities. Interventions must be seen as the sum of altering multiple phenotypic axes that modulate innate and adaptive immunity as well as host tissue susceptibility.

Several challenges in the field remain. From a mechanistic standpoint, the immunologic impact of the donor-derived microbiota on donor graft T-cell responsiveness, as well as subsequent reactivity to the recipient microbiota, remains largely unexplored. From a translational perspective, defining donor and recipient characteristics that maximize FMT efficacy and optimizing defined commensal consortia to improve infectious and GvHD outcomes would substantially advance microbiota-based therapeutic strategies. Additional clinical data are also needed to determine whether prebiotic approaches can reliably expand beneficial commensal anaerobes and meaningfully impact transplant outcomes, particularly as an alternative to direct ecosystem replacement. Ultimately, by emphasizinga microbiota-integrated model of GvHD, the field is poised to move beyond reactive damage control and toward therapies that proactively shape the immunologic and epithelial environment that govern GvHD and transplant outcomes.

## Supplementary Material

Supplementary material Therapeutics Supplemental Table 1.csv
